# Aland Island Eye Disease with Retinoschisis in the Clinical Spectrum of *CACNA1F*-Associated Retinopathy—A Case Report

**DOI:** 10.3390/ijms25052928

**Published:** 2024-03-02

**Authors:** Dorota Wyględowska-Promieńska, Marta Świerczyńska, Dorota Śpiewak, Dorota Pojda-Wilczek, Agnieszka Tronina, Mariola Dorecka, Adrian Smędowski

**Affiliations:** 1 Department of Ophthalmology, Faculty of Medical Sciences in Katowice, Medical University of Silesia, 40-514 Katowice, Poland; 2 Kornel Gibiński University Clinical Center, 40-514 Katowice, Poland; 3 Department of Pediatric Ophthalmology, Faculty of Medical Sciences in Katowice, Medical University of Silesia, 40-514 Katowice, Poland; 4 GlaucoTech Co., 40-282 Katowice, Poland

**Keywords:** Aland island eye disease, AIED, CACNA1F, Cav1.4, retinoschisis

## Abstract

Aland island eye disease (AIED), an incomplete form of X-linked congenital stationary night blindness (CSNB2A), and X-linked cone-rod dystrophy type 3 (CORDX3) display many overlapping clinical findings. They result from mutations in the *CACNA1F* gene encoding the α_1F_ subunit of the Cav1.4 channel, which plays a key role in neurotransmission from rod and cone photoreceptors to bipolar cells. Case report: A 57-year-old Caucasian man who had suffered since his early childhood from nystagmus, nyctalopia, low visual acuity and high myopia in both eyes (OU) presented to expand the diagnostic process, because similar symptoms had occurred in his 2-month-old grandson. Additionally, the patient was diagnosed with protanomalous color vision deficiency, diffuse thinning, and moderate hypopigmentation of the retina. Optical coherence tomography of the macula revealed retinoschisis in the right eye and foveal hypoplasia in the left eye. Dark-adapted (DA) 3.0 flash full-field electroretinography (ffERG) amplitudes of a-waves were attenuated, and the amplitudes of b-waves were abolished, which resulted in a negative pattern of the ERG. Moreover, the light-adapted 3.0 and 3.0 flicker ffERG as well as the DA 0.01 ffERG were consistent with severely reduced responses OU. Genetic testing revealed a hemizygous form of a stop-gained mutation (c.4051C>T) in exon 35 of the *CACNA1F* gene. This pathogenic variant has so far been described in combination with a phenotype corresponding to CSNB2A and CORDX3. This report contributes to expanding the knowledge of the clinical spectrum of *CACNA1F*-related disease. Wide variability and the overlapping clinical manifestations observed within AIED and its allelic disorders may not be explained solely by the consequences of different mutations on proteins. The lack of distinct genotype–phenotype correlations indicates the presence of additional, not yet identified, disease-modifying factors.

## 1. Introduction

Aland island eye disease (AIED; OMIM #300600) is a rare X-linked recessive disorder of unknown incidence. The phenotype in affected males is highly variable and comprises nystagmus, myopia, astigmatism, reduced visual acuity, protan color vision defect, impaired dark adaptation, iris trans-illumination, fundus hypopigmentation, foveal hypoplasia, as well as shorter foveal cone outer segment lengths and subfoveal choroidal thinning. Besides the progression of axial myopia, the disease presents as a stable condition [1,2,3]. Although AIED was initially considered a variant of X-linked ocular albinism, the occurrence of latent nystagmus of extraocular origin, no evidence of optic fiber misrouting and the absence of macromelanosomes in skin exclude this concept [4,5,6]. Female carriers usually do not present with abnormalities, although in some cases, there have been reports of slight latent nystagmus, mild to moderate loss of vison or electroretinography (ERG) abnormalities [5,7].

The *CACNA1F* (MIM 300110; calcium channel, voltage-dependent, alpha-1F subunit) gene consists of 48 exons spanning a region of 28 kb in the pericentromeric region of the Xp11.23 chromosome [2]. It encodes the α_1F_ subunit of the L-type voltage-gated calcium channel Cav1.4 (LTCC), which is primarily expressed in both rod and cone terminals. These channels mediate the sustained influx of Ca^2+^ into the cell, upon which depends the continuous release of glutamate at the ribbon synapses of the retinal photoreceptors in the dark [8,9,10]. Pathogenic alterations of the above ion channels consequently result in a reduction in signal transfer and this electrophysiological hallmark is the incomplete Schubert–Bornschein type of ERG, in which the amplitude of the scotopic b-wave is smaller than the a-wave, and photopic responses are markedly affected [3,10,11].

Mutations in the *CACNA1F* gene can be associated with non-progressive diseases (AEID, incomplete form of X-linked congenital stationary night blindness (CSNB2A; OMIM #300071)), as well as progressive conditions including X-linked cone-rod dystrophy type 3 (CORDX3; OMIM #300476), retinitis pigmentosa or early-onset high myopia [2,12,13]. There are 346 pathogenic *CACNA1F* variants described to date, and the most common phenotype is CSNB2A, which shows many overlapping clinical features with AIED: nystagmus, astigmatism, decreased visual acuity and impaired night vision. These channelopathies display similar electroretinographic changes that indicate a loss of neurotransmission from photoreceptors to bipolar cells as well as varying degrees of cone and rod impairments. Regarding the differences between AIED and CSNB2A, the former exhibits additional progressive myopic refraction, a protan defect in color vision, iris trans-illumination, a hypopigmented fundus and foveal hypoplasia [1,2,3,10,11]. However, CORDX3, unlike the previous two congenital disorders, can begin in adulthood, and is characterized by a progressive nature concerning refraction, visual acuity, color vision and visual fields (central scotoma is frequently present). The appearance of the fundus can vary from normal through subtle granularity of the macula up to bull’s eye maculopathy or central retinal pigment epithelium atrophy. There is no nystagmus, no astigmatism above 1.5 D and, unlike CSNB2A, no hyperopic refraction [13]. Significant clinical variability and phenotype overlapping suggest that AIED, CSNB2A and CORDX3 may represent a broad continuous spectrum of the same clinical disease.

Here, we present a 57-year-old patient with AIED and retinoschisis with a hemizygous form of a stop-gained variant (c.4051C>T) in exon 35 of the *CACNA1F* gene. This report contributes to expanding the knowledge of the phenotypic spectrum of *CACNA1F*-related disease.

## 2. Case Report

A 57-year-old Caucasian man, who had suffered from nystagmus, nyctalopia, low visual acuity and progressive high axial myopia in both eyes (OU) since early childhood, visited the Outpatient Ophthalmology Clinic to expand the diagnostic process. This was due to the onset of similar symptoms in his 2-month-old grandson including horizontal nystagmus and photophobia. Further ophthalmologic examination of the infant revealed the presence of myopia and astigmatism, as well as trans-illumination of the iris, hypopigmentation of the fundus and foveal hypoplasia OU. There was no family history of consanguinity and his past medical history was unremarkable; similar symptoms were not present in the boy’s mother or among other family members.

**Figure 1:**
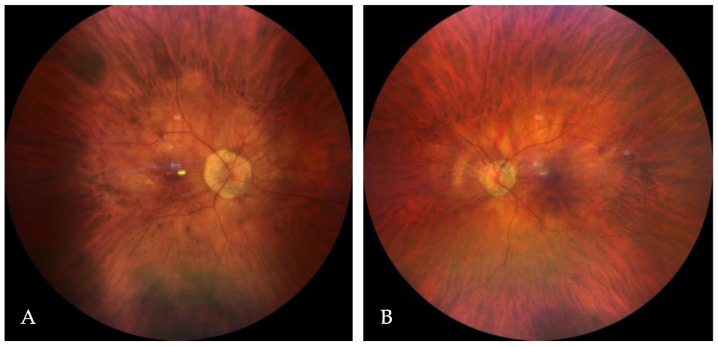
The color fundus images of the right (**A**) and the left (**B**) eye.

**Figure 2:**
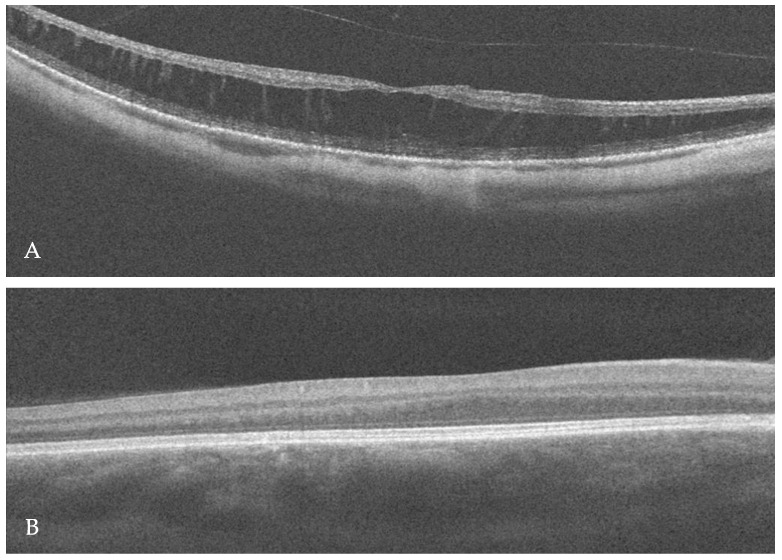
Optical coherence tomography (OCT) scan of the right macula revealed retinoschisis (**A**), whereas foveal hypoplasia was visible in the left eye (**B**).

In this patient, the best-corrected visual acuity (BCVA) was 0.2 in the right eye (RE) and 0.3 in the LE (LE). The refraction of the RE was −12.0 diopters of spherical power (DS)/−0.25 diopters of cylinder power (DC) × 30° and of the LE was −7.25 DS/−0.75 DC × 170°. Color vision testing showed a protanomalous color vision deficit. Horizontal jerk nystagmus without compensatory head alignment was present. An anterior segment examination revealed posterior subcapsular cataract OU (more advanced in the RE). However, no trans-illumination of the iris was observed. An examination of the eye fundus demonstrated a slightly pale optic disc surrounded by an atrophic area, diffuse thinning, and moderate hypopigmentation of the retina with visible choroidal vasculature OU (Figure 1A,B). Fundus autofluorescence (FAF) imaging (Spectralis HRA, Heidelberg Engineering, Heidelberg, Germany) of the posterior pole was within the normal limit OU, whereas optical coherence tomography (OCT) (Avanti Widefield, Optovue, Fremont, CA, USA) revealed retinoschisis in the RE and foveal hypoplasia in the LE (Figure 2A,B).

**Figure 3:**
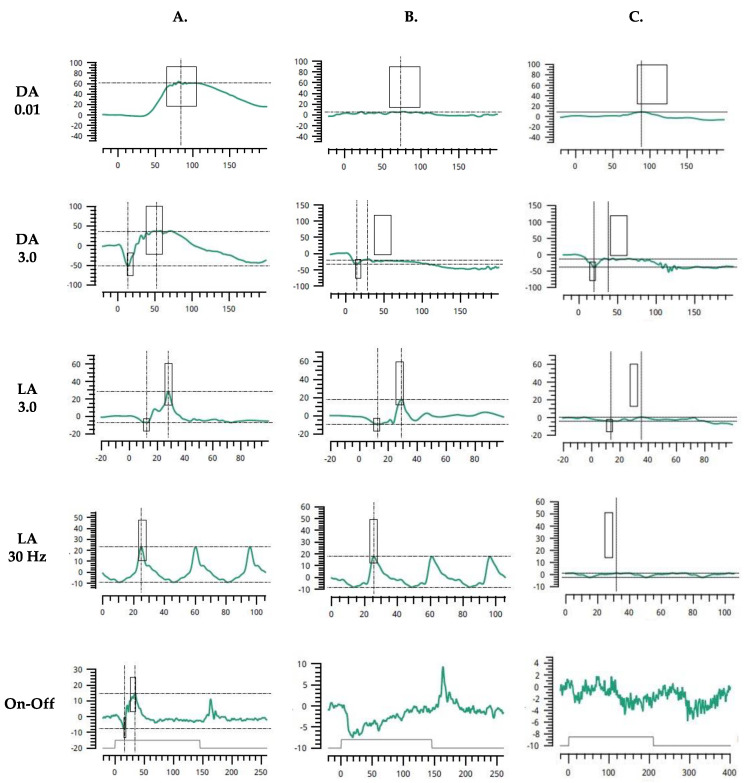
Flash full-field electroretinogram (ffERG) characteristics in control subject (**A**); patient with CSNB type 1, complete (CSNB1) (**B**); index patient with Aland Island eye disease (AEID) (**C**). DA 0.01—dark-adapted 0.01 ERG (rod response); DA 3.0—dark-adapted 3.0 ERG (combined rod-cone response); LA 3.0—light-adapted 3.0 ERG (single-flash cone response); LA 3.0 flicker—light-adapted 3.0 flicker ERG (30 Hz flicker, cone response); On-Off—On-Off ERG (bipolar cell response).

Visual evoked potentials (VEPs) were assessed with the RETI-port/scan21 unit (Roland Consult, Wiesbaden, Germany) in accordance with the standards of the International Society for Clinical Electrophysiology of Vision (ISCEV) [14]. Examinations revealed low amplitudes and delayed latencies of the P100-waves in pattern VEPs (PVEPs) and P2-waves in flash VEPs (FVEPs). There was no inter-hemispheric difference when each eye was tested independently. Flash full-field electroretinography (ffERG) was tested, using the RETeval portable device (LKC, USA) and sensor strip electrodes, in accordance with the standards of the ISCEV [15]. The light-adapted (LA) 3.0 ffERG (single-flash cone response) and LA 3.0 flicker ffERG (30 Hz flicker) were consistent with severely reduced amplitudes OU. Likewise, the isolated rod response in dark-adapted (DA) 0.01 ffERG was almost completely suppressed bilaterally. Moreover, in DA 3.0 ffERG assessing the combined rod–cone response, the amplitudes of the a-waves were about 10.–20. percentiles (normal range from −22.0 μV to −79.0 μV) and the amplitudes of the b-waves were abolished, which results in a negative pattern of the ERG. On-Off ffERG protocols revealed unmeasurable function of both the ON and OFF cone bipolar pathways (Figure 3A–C).

**Figure 4:**
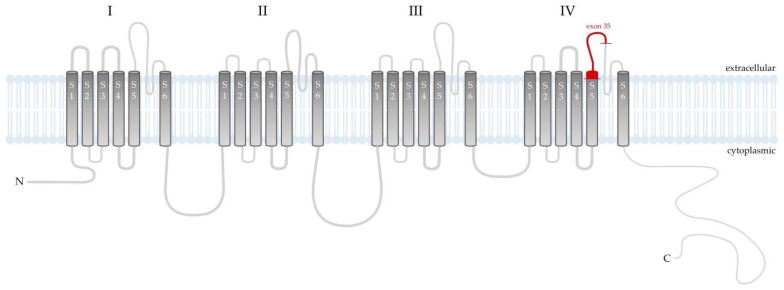
Schematic presentation of the Cav1.4 channel that is composed of a main pore-forming α_1_ subunit and auxiliary β and α_2_δ_4_ subunits. Cav1.4α_1_ subunit is a single peptide chain composed of four similar domains (**I**–**IV**), each repeat contains six transmembrane helices (named S1–S6) linked by intracellular loops between the S5 and S6 segments. Helices S1–S4 of each repeat act as the voltage sensing domain (VSD). Helices S5–S6 of the four repeats with their connecting linker comprise the ion-conducting pore, which permit ions to flown down the electrochemical gradient from the extracellular milieu into the cytoplasm. In the distal end of the C-domain, the autoinhibitory domain is located [16]. In the index patient, the mutation involved the extracellular region between IV-S5 and IV-S6 and an insertion of a premature stop codon altered membrane topology for the C-terminal part of the protein.

Based on the constellation of clinical symptoms and diagnostic test results in the maternal grandfather and his grandchild, a sequence analysis of the coding regions of 24 genes (*AP3B1*, *AP3D1*, *BLOC1S3*, *BLOC1S5*, *BLOC1S6*, *CACNA1F*, *DTNBP1*, *GPR143*, *HPS1*, *HPS3*, *HPS4*, *HPS5*, *HPS6*, *LRMDA*, *LYST*, *MC1R*, *MITF*, *OCA2*, *PAX3*, *RAB27A*, *SLC24A5*, *SLC45A2*, *TYR*, *TYRP1*) whose pathogenic variants correlate with the clinical manifestations of albinism and hypopigmentation was carried out. A next-generation sequencing (NGS) method was performed using a SeqCap EZ HyperCap Workflow and NimbleGen SeqCap EZ Library Kit (Roche, Basel, Switzerland). The NextSeq 1000 instrument (Illumina Inc., San Diego, CA, USA) was used for this study. Meanwhile, using the Sanger sequencing technique, the presence of the hemizygous form of the NM_001256789.3:c.4051C>T mutation in exon 35 of the *CACNA1F* gene, resulting in premature translation termination (NP_001243718.1:p.Arg1351Ter) (Figure 4), was confirmed. This nonsense variant is classified in the Human Gene Mutation Database as pathogenic, which in conjunction with the abnormalities present in both patients resulted in the diagnosis of AIED. Unfortunately, the patient’s daughter did not decide to have a genetic test.

## 3. Discussion

The *CACNA1F* gene encodes a main pore-forming α_1F_ subunit of the Cav1.4 channel, which, in the human retina, exerts expression in the outer plexiform layer, inner nuclear layer, inner plexiform layer, and nerve fiber layer [8]. Ultimately, in the dark, the photoreceptor membrane potential depolarizes, inducing a sustained influx of Ca^2+^ through the LTCC, which in turn enhances tonic glutamate release. The signal is then transmitted to the bipolar cells, which also show expression of the above ion channels and participate in the regulation of the release of neurotransmitters [9]. The Cav1.4 channel is tailored for this function, as it has a fast activation, enables Ca^2+^ influx at relatively negative voltages, and undergoes a slow voltage-dependent inactivation in the absence of Ca^2+^-dependent inactivation (CDI) [9,17,18]. Moreover, Cav1.4 contributes to the maturation of photoreceptor ribbon synapses and acts as a synaptic organizer protein [19,20].

Malfunctioning Cav1.4 impairs transmission from rod and cone photoreceptors to bipolar cells and is responsible for significantly reduced b-waves, leading to a negative pattern in DA 3.0 ERG. In CSNB2, DA ERG responses are diminished but recordable, whereas LA ERG findings are more impaired. The dysfunction in both ON and OFF pathway activity is also present. These features distinguish it from complete CSNB (CSNB1), in which photopic cone function is less impaired and ON bipolar cells are selectively affected, whereas rod responses are more attenuated [3,10,11]. A significant reduction in flash ffERG responses in the index patient suggests a complete absence of functional Cav1.4 channels but may be further enhanced by the co-occurrence of high myopia, which also contributes to the reduction in the a- and b-wave amplitude [21].

Retinoschisis was found in the abovementioned patient, and it probably developed as a result of mechanical traction at the vitreomacular interface in association with myopia [22]. However, an interesting point is that there has been demonstrated a bidirectional relationship between LTCCs (Cav1.3 and Cav1.4) and retinoschisin (RS1). In mouse retinas, the deletion of Cav.1.4 leads to a decrease in RS1, and vice versa [23]. RS1 is an adhesion and anchoring membrane protein secreted from photoreceptors and bipolar cells, playing an important role in maintaining the stability of retinal layers, while mutations of the *RS1* gene are associated with X-linked juvenile retinoschisis [24]. With Cav1.4 deletion, a diminution of Ca^2+^ influx through LTCCs may reduce the expression or secretion of RS1 from photoreceptors. Similarly, impaired Cav1.4 function can lead to structural defects and/or a reduction in the number of ribbon synapses, resulting in a concomitant decrease in the amount of RS1, which is essential for binding ion channels [25,26]. Moreover, the genes encoding RS1 and Cav1.4, located on the X chromosome, can mutually affect their expression. Therefore, it is possible that in the case of mutations in *CACNA1F*, not only myopia-induced abnormalities but also a subsequent lack of RS1 may impede the maintenance of the structural stability of the retina.

A wide range of phenotypic variability within *CACNA1F*-related diseases is noted. An analysis of 66 patients with CSNB2A (sharing the same mutation: 3166–3167insC) found the absence of at least one of the main symptoms (myopia, nystagmus, impaired dark adaptation) in 72% of the individuals examined, with 40% of the subjects reporting no night vision problems. Significant intra- and interfamilial variations in the clinical manifestations were also observed [27]. The high variability and symptom overlap makes it impossible to differentiate between CSNB2A and AIED in some patients [12]. The nonsense mutation detected in our patient, c.4051C>T in *CACNA1F*, according to the ClinVar Database, has so far been reported in association with CSNB2A and CORDX3. However, in the index case, its presence in conjunction with the constellation of clinical symptoms and diagnostic test results led to the diagnosis of AIED.

According to Mihalich et al. [28], different clinical phenotypes may depend on the location of the mutations in the *CACNA1F* gene. Presumably, the symptoms would be more severe when the mutation affects the first half of the protein and as a result of a significant disruption of its structures; there is a complete lack of these channels. However, mutations involving the more terminal part of the protein may confer less expressed alterations in channel function. As an example, the authors mention mutations in exon 4 resulting in an AIED-like phenotype, and in exon 43, which are associated with less severe symptoms associated with CSNB2A. In the latter case, the channel probably was positioned in the membrane correctly but had a hyperpolarized shift in activation.

Cav 1.4 channels lack CDI due to active suppression by the inhibitory domain in their C-terminus, a phenomenon referred to as C-terminal modulation (CTM) [18]. The CTM not only determines CDI, but also affects the activation gate properties of the channel and the likelihood of opening. Mutations affecting the CTM will not support continuous Ca^2+^ influx and might thereby reduce the dynamics of photoreceptors [29]. However, there is a lack of data from in vivo models that can specify which processes dominate when CTM function is affected [10]. However, in our patient, the mutation c.4051C>T in exon 35 of the *CACNA1F* gene was responsible for a change (localized in the extracellular region between IV-S5 and IV-S6) leading to the insertion of a premature stop codon, preventing the translation of the remaining part of the protein. This alteration in protein topology and significantly disturbed ERG responses suggest a complete absence of the Cav1.4 channel or loss of its function.

The detection of a novel p.Gly603Arg mutation in the *CACNA1F* gene, which in the proband resulted in AIED while in his maternal grandfather resulted in CSNB2A, suggests that these disorders are not mutation specific and may not be distinct [30]. Conversely, there are a few reports of an atypical *CACNA1F*-associated phenotype. One example is the Maori family, where severe CSNB2-like phenotype was associated with intellectual impairment among male patients, while all female carriers manifested evident electrophysiological and clinical abnormalities (nystagmus, decreased visual acuity, high myopia) [31]. Moreover, the same mutation found in siblings with retinal and optic disc atrophy and progressive deterioration of vision resulted in clinical and ERG findings typical of CSNB2A in other patients [32]. These findings prove that the diversity of AIED and CSNB2A phenotypes is not solely a consequence of intragenic allelic heterogeneity. Differences in genetic background between other mutations or polymorphisms in other genes may be relevant, so that acting synergistically with variants in the *CACNA1F* gene may induce additional pathological changes. Additionally, environmental factors found both before and/or after birth may play an important role [30,32]. Given the significant phenotype overlap, many studies suggest that AIED and CSNB2A or AIED, CNSB2A, and CORD3 are a broad continuous spectrum of the same clinical disease. It has been suggested that the diseases should be classified by genotype, naming them *CACNA1F*-associated retinopathy.

Currently, there is no gene therapy available for *CACNA1F*-associated retinopathy. It has been postulated that Cav1.4 mutations might be repaired in the photoreceptor genome via the use of zinc finger nucleases, TALEN, or CRISPR/Cas as molecular tools [33,34,35]. Waldner et al. [36] created a mouse line that showed the partial structural and functional rescue of retinal integrity through cre-induced expression of a transgenic Cav1.4. Cre expression was controlled by the Pax6 promoter, resulting in Cav1.4 expression in the early development stage. In contrast, Laird et al. [37] used a tamoxifen-induced promoter to temporarily express transgenic Cav1.4. Rods were subjected to transfection with plasmids carrying the transgene via in vivo retinal electroporation on the day of birth. In both young and mature animals, the induction of Cav1.4 expression partially salvaged the synaptic features. However, technical difficulties were demonstrated due to the retinal detachment observed in the tamoxifen-induced group.

However, it is important to note the limitations of the above publication, which is based on the description of a single family. It is important to remember that potential confounding factors (e.g., environmental factors, other genetic modifiers), patient-specific factors, the lack of a long follow-up period, incomplete genetic analysis, as well as ethnic and/or geographic differences in *CACNA1F*-related disease manifestation may result in a clinical presentation of our patients that may not fully represent the variability of the disease phenotype and may not be relevant to the larger population with similar genetic conditions. Therefore, conclusions based on the above publication should be made with caution.

## 4. Conclusions

The variability of clinical manifestations observed in AIED and its allelic disorders CSNB2A as well as CORDX3 cannot be solely explained by the consequences of different mutations in the *CACNA1F* gene on protein function, and this report contributes to expanding the knowledge of the clinical spectrum of *CACNA1F*-related disease. The absence of clear genotype–phenotype correlations indicates the presence of additional disease-modifying factors. Understanding the relationship that causes the clinical variability associated with pathogenic *CACNA1F* variants may be useful in both clinical and molecular genetic diagnostics and may potentially assist in the development of more specific therapeutic strategies.

## Data Availability

Data are contained within the article.
